# Chitosan and its derivatives as vehicles for drug delivery

**DOI:** 10.1080/10717544.2017.1399305

**Published:** 2017-11-10

**Authors:** Gangliang Huang, Yang Liu, Ling Chen

**Affiliations:** Active Carbohydrate Research Center, Chongqing Key Laboratory of Inorganic Functional Materials, College of Chemistry, Chongqing Normal University, Chongqing, China

**Keywords:** Chitosan, derivatives, vehicles, sustained release, controlled release, targeted drug delivery

## Abstract

Chitosan and its derivatives as vehicles for drug delivery can achieve the purpose of sustained release and controlled release for drugs, improve the stability of drugs, and reduce adverse drug reactions. So, the bioavailability of drugs can be enhanced. Therefore, chitosan and its derivatives have become a hotspot in the field of drug delivery. Their characteristics as drug delivery vectors were introduced, the types and applications were summarized. The development direction of chitosan and its derivatives in this field was also forecasted.

## 1. Introduction

Chitosan is a kind of natural polysaccharide cellulose, widely found in insect, crustacean shell and fungal cell wall, also known as soluble chitin, which is nontoxic, biocompatible, biodegradable, etc. (Riva et al., 2011). Its preparation is simple, source is rich, and the hydrophilicity is strong. Chitosan can be biodegradable by *in vivo* lysozyme, pepsin, and other enzymes. The degradation products are nontoxic and can be completely absorbed by the organisms. Moreover, chitosan has the ability to resist acid, anticoagulant, and ulcer. It can prevent or weaken the stinging reaction caused by drugs in the stomach. In addition, the chitosan matrix can form floating and gradually expand in acidic environment. These characteristics make chitosan an ideal drug delivery material (Sonia & Sharma, 2011).

The vehicle of nano-sized polymer particle for drug delivery and controlled release, because of its ultra-small size, reasonable *in vivo* distribution and efficient drug utilization, can pass the tissue epithelium. So, the targeted delivery and controlled release of drugs are more effective. Therefore, the nano-sized polymer particle becomes the ideal vehicle of embedded peptide, protein, nucleic acid, vaccine, and other bioactive macromolecules (Lee et al., 2010).

Chitosan has good film-forming property; the film has good biological compatibility and permeability. It has important development and research value in sustained-release drugs and targeted drug delivery. So, chitosan is often used as vehicle for the sustained release of drugs. Many drugs are administered through mouth, nose, and mucous membrane. The drugs are released slowly over a long period of time, so as to achieve the goal of sustained administration (Mengatto et al., 2012).

Chitosan has a unique polycationic property that can interact with sodium alginate (polyanion) by electrostatic interaction. The sodium alginate microcapsule surface is attached with a layer of polyelectrolyte membrane. Thereby, it can improve the stability of microcapsules and drug loading, and adjust the drug release rate. The higher the chitosan content is in the microcapsules, the stronger the sustained release effect of sodium alginate is (Lam et al., 2012). The sustained release effect in the buffer with pH 4 ∼ 5 is significantly greater than that in the buffer with pH 1 ∼ 2. The results of study can be used for the dosage form design of drugs with high gastric irritation.

Chitosan is a hydrophilic polymer, which can be used to prepare microspheres with different sizes. The combination of microspheres and drugs can avoid the use of organic solvents, and prevent the denaturation of antigenic proteins. Chitosan microspheres have porous structure, which makes antigen proteins not only adsorbed on the surface of microspheres, but also can be embedded into the inner surface of chitosan microspheres. Chitosan microspheres can also control the release of protein drugs and enhance the absorption of antigen protein (Saravana & Ramaswamy, 2011). In view of the stability of proteins, most researchers prepared chitosan blank microspheres, and then passively adsorbed antigen to microspheres.

Chitosan is a nontoxic and biocompatible polycation with low immunity. It is a good choice for gene delivery systems, because chitosan has cations that can bind effectively to DNA with anions and protect it from nuclease degradation. Chitosan particles loaded with DNA are stable in storage, so it as a vehicle of gene drugs is of great significance (Saranya et al., 2011).

Hydrogels have a 3-D network structure of cross-linked hydrophilic polymer, and have attracted much attention because of their potential uses in a variety of therapeutic and biomedical applications (Bhattarai et al., 2010).

Herein, chitosan and its derivatives as vehicles, such as nanoparticles, microcapsules, microspheres, hydrogels, and so on, for delivery of chemical drugs and genes or proteins were summarized and discussed.

## 2. Chitosans and its derivatives as vehicles to deliver chemical drugs

Chitosan nanoparticle as a novel drug vehicle has nontoxicity, good biocompatibility, biodegradability, improving drug stability, changing the route of administration, increasing drug absorption, improving the bioavailability of drugs, and other characteristics. So, it can achieve the role of controlled release and targeted therapy for drugs *in vivo* (Alishahi et al., 2011). According to the structural characteristics of chitosan and the nature of drug itself, drug-loaded chitosan nanoparticles can be prepared by a variety of methods. Chitosan has become the focus of research on targeted and slow/controlled release, and has broad application prospects. In addition, the surface modification of chitosan nanoparticles, which has the selectivity required by target organs, target tissues and target cells (Bhargava et al., 2015), can be carried out by using modern molecular design ideas and advanced synthesis techniques. It is also the development trend of future research.

Khodadust et al. (2014) produced different sized chitosan MNPs by *in situ* synthesis method. It showed that the chitosan MNPs had pH responsive release characteristics. Doxorubicin (DOX)-loaded chitosan MNPs were efficiently taken up by MCF-7 (MCF-7/S), and DOX was resistant to MCF-7 (MCF-7/1 μM) breast cancer cells, which increased the efficacy of drug and also maintained overcoming the resistance of DOX in MCF-7/DOX cells. So, chitosan MNPs, which were synthesized at various sizes, could be effectively used for the pH dependent release of DOX in cancer cells. Therefore, it can provide new insights in the development of pH responsive targeted DDSs to overcome the side effects of conventional chemotherapy.

Tamoxifen (Tam) has a broad spectrum of anticancer activity, but it is limited in clinical application. Vivek et al. (2013) investigated the smart pH-responsive drug delivery system (DDS), which was based on chitosan nanoparticles for their potential in enabling more intelligent controlled release and enhancing chemotherapeutic efficiency of Tam. Tam was loaded onto chitosan-nanoparticles by forming complexes and it was released from the DDS much more rapidly at pH 4.0 and 6.0 than at pH7.4, which is a desirable characteristic for tumor-targeted drug delivery. It was demonstrated that Tam-loaded chitosan nanoparticles increased intracellular concentration of Tam in human breast cancer MCF-7 cells and enhanced their anticancer efficiency by inducing apoptosis in a caspase-dependent manner, which indicated that drug-loaded nanoparticles could act as an efficient DDS importing Tam into target cancer cells.

Termsarasab et al. (2014) successfully synthesized chitosan oligosaccharide-arachidic acid (CSOAA) conjugate, which was used to develop self-assembled nanoparticles for DOX delivery. It indicated that DOX-loaded CSOAA-based nanoparticles exhibited the sustained and pH-dependent drug release profiles according to the result of *in vitro* release test. The CSOAA showed negligible cytotoxicity in FaDu (human head and neck cancer) cells. Cellular uptake of DOX in FaDu cells was higher in the nanoparticle-treated group compared to the free DOX group. The antitumor efficacy of DOX-loaded nanoparticles was also verified in FaDu tumor xenografted mouse model. These results suggested that synthesized amphiphilic CSOAA might be used to prepare the self-assembled nanoparticles for anti-cancer drug delivery.

Acyclovir (ACV) is one of the drugs of choice to treat epidermal, ocular, or systemic herpetic infections. However, its trans-mucosal limited absorption and the scarce contact time of the formulation with the mucosal surface (especially in the ocular mucosa) constitute a big limitation of the antiviral efficiency. The most effective way to solve the above problems is to increase the quantity and the residence time of the drug over the ocular surface. The chitosan nano- and microparticles (MPs) could be used for topical drug delivery (Sezer & Cevher, 2012). In order to meet all these requirements, Calderón *et al.* (Calderón et al., 2013) developed MPs and nanoparticles containing ACV using cross-linked chitosan with tripolyphosphate owing to the biocompatibility, bio-adhesion ability and the potential power as penetration enhancer of this polymer. It indicated that the amounts of ACV effectively diffused in 24 h were 30, 430, and 80 μg for the ACV solution, MP and nanoparticle, respectively. It also showed that chitosan-based particles induced moderate irritation and mild tissue damage from slug mucosal irritation (SMI) results, what supposes that ACV-MP constitutes a promising alternative for further development of an antiviral formulation.

Shen et al. (2012) used the novel carboxymethyl chitosan-based folate/Fe_3_O_4_/CdTe nanoparticle to achieve targeted drug delivery and cell imaging. Grenha et al. (2010) develop the new chitosan/carrageenan nanoparticles to deliver drugs. Feng et al. (2013) utilized the chitosan/*O*-carboxymethyl chitosan nanoparticles to efficiently and safely deliver the oral anticancer drug. Zhou et al. (2013) prepared the galactosylated chitosan-polycaprolactone nanoparticles for hepatocyte-targeted curcumin delivery. Anitha et al. (2012) used the curcumin-loaded *N*,*O*-carboxymethyl chitosan nanoparticles to deliver cancer drug. Millotti et al. (2011) used chitosan-6-mercaptonicotinic acid nanoparticles to deliver oral peptide. Xing (2013) prepared the folate modified-chitosan nanoparticles for new tumor-targeting drug delivery.

Pengpong et al. (2014) designed and prepared a hydrophobic mucoadhesive thiolated chitosan for hydrophobic drug delivery by conjugating *p*-coumaric acid (pCA) to increase hydrophobic compatibility with drug *via* pi-pi interaction and then covalently linking homocysteine thiolactone (HT) to the pCA-chitosan to increase the mucoadhesive properties. A model hydrophobic drug, piperine (PIP), was encapsulated in pCA-HT-chitosan MPs *via* electrospray ionization with an encapsulation efficiency of over 80%. It showed that a sustained release of PIP was >75% over 12 h between pH 1.2 and 6.4 from *in vitro* release experiments.

Chitosan microspheres show some unique functional advantages. For example, they have rich polysaccharide chains, which can be identified by specific cells or tissues. Chitosan microspheres can target the delivery of drugs to the lesion site for storage and release (Mei et al., 2009). Their surface can be grafted with functional groups, which can be flexibly loaded with different drugs by adsorption or wrapping (Varshosaz, 2007). Based on the above advantages, chitosan microspheres have become a hotspot in recent years.

Abdel Mouez et al. (2014) produced the microspheres using a spray-drying and precipitation techniques. It was demonstrated that the microspheres were spherical with size 21-53 μm suitable for nasal deposition. It indicated that the nasal microspheres exhibited a significantly higher bioavailability (58.6%) than nasal solution of verapamil hydrochloride (VRP) (47.8%) and oral VRP solution (13%) from the bioavailability study. Overall, the chitosan-based nasal VRP microspheres are promising to enhance VRP bioavailability by increasing the nasal residence time and avoiding the first-pass metabolism of the drug substance.

Patil & Sawant (2011) developed and characterized chitosan mucoadhesive microspheres for nasal delivery. They prepared microspheres by emulsification-crosslinking method. It showed that almost entire amount was delivered after three puffs from the results of powder delivery. It was demonstrated that microspheres were delivered forming an elongated puff from the images of delivery sequences of microsphere powder clouds. The core of the clouds was homogeneous, which could be expected to offer effective distribution pattern.

Kawadkar et al. (2013) prepared genipin cross-linked chitosan microspheres of flurbiprofen for intra-articular (i.a.) delivery. The microspheres were prepared using different concentrations of genipin and drug-to-polymer ratios by emulsion-cross-linking method. The optimized microspheres could release the drug for more than 108 h. The recovery of flurbiprofen as the percent of administered dose followed by 24 h after i.a. injection of microspheres was found to be 8.7 folds higher than its solution.

Wang et al. (2010) used calcium carbonate/carboxymethyl chitosan hybrid microspheres and nanospheres for drug delivery. Umadevi et al. (2010) prepared the chitosan microspheres of aceclofenac for colon-targeted drug delivery. Zhang et al. (2013) developed a novel phytosome-loaded chitosan microsphere for the delivery of curcumin. Xing (2013) prepared the folate-decorated chitosan microspheres for sustained release drug delivery.

Gratieri et al. (2011) evaluated the potential of a chitosan solution and an *in situ* gel-forming system, which was comprised of poloxamer/chitosan as vehicles for enhanced corneal permeation and sustained release of fluconazole (FLU). It showed that the sustained release of FLU was observed from the poloxamer/chitosan formulation by the *in vitro* release studies. It was demonstrated that the formulations studied had a permeation-enhancing effect by *ex vivo* permeation studies across porcine cornea, which was independent of chitosan concentration in the range from 0.5 to 1.5% w/w. It showed that there was the greatest *ex vivo* drug permeation for the chitosan solutions alone. However, the poloxamer/chitosan formulation presented similar *in vivo* performance than the chitosan solution at 1.0%; both formulations showed sustained release and about 3.5-fold greater total amount of FLU permeated when compared with simple aqueous solutions of the drug. In a word, it was demonstrated that both the *in situ* gelling formulation evaluated and the chitosan solution are viable alternatives to enhance ocular bioavailability in the treatment of fungal keratitis.

Wang et al. (2013) developed a near infrared (NIR) triggered drug delivery platform, which was based on the chitosan-modified chemically reduced graphene oxide (CRGO) incorporated into a thermosensitive nanogel (CGN). CGN exhibited an NIR-induced thermal effect, which was similar to that of CRGO, it had reversible thermo-responsive characteristics at 37-42 °C and high DOX hydrochloride (DOX) loading capacity (48 wt%). The DOX-loaded CGN (DOX-CGN) released DOX faster at 42 °C than at 37 °C. It revealed that DOX expression was in the cytoplasm of cancer cells when incubated with DOX-CGN at 37 °C but in the nucleus at 42 °C by fluorescence images. Upon irradiation with NIR light (808 nm), a rapid and repetitive DOX release from the DOX-CGN was observed. Moreover, the cancer cells incubated with DOX-CGN and irradiated with NIR light displayed significantly greater cytotoxicity than without irradiation because of NIR-triggered increase in temperature leading to nuclear DOX release. Therefore, these results demonstrate CGN's promising application for on-demand drug release by NIR light.

Chitosan as a natural polymer in the application of hydrogels, has attracted great attention in recent years. Because it has good biocompatibility, low toxicity, and can be degraded by enzymes in the human body, coupled with its unique physical characteristics (such as hydrophilicity, functional amino acid group and cationic net charge), this makes chitosan a perfect tool for intelligent drug delivery (Rani et al., 2010).

Chitosan-based thermosensitive solutions, which turn into semi-solid hydrogels upon injection at body temperature, have increasingly drawn attention over the last decades as an attractive new type of *in situ* forming depot (ISFD) DDS. Supper et al. (2014) investigated glucose-1-phosphate (G1-P) as an alternative gelling agent for improving the stability of chitosan-based ISFD solutions. It showed that a sustained release was over days to weeks for hydrophilic model compounds from *in vitro* release experiments, which demonstrated thereby that chitosan/G1-P might be suitable for the prolonged delivery of drugs. The inflammatory reaction observed in the tissue surrounding the hydrogel in rats was a typical foreign body reaction. It confirms the potential of chitosan/G1-P solutions as an injectable ready-to-use *in situ* forming hydrogel from these features.

Lee et al. (2011) prepared decanoic acid-modified glycol chitosan (DA-GCS) hydrogels containing tightly adsorbed palmitic acid-modified exendin-4 (Ex4-C16). GCS was conjugated with *N*-hydroxysuccinimide-activated DA in anhydrous 0.4% dimethylaminopyridine/dimethylsulfoxide at different feed ratios. DA-GCS hydrogels were formed by physical self-assembly during dialysis vs. deionized water. The hypoglycemia caused by Ex4-C16-loaded DA-GCS hydrogels was evaluated by subcutaneous administration in type 2 diabetic *db*/*db* mice. The *in vitro* and *in vivo* release of Ex4-C16 from DA-GCS hydrogels was dramatically delayed compared with native Ex4 probably due to strong hydrophobic interactions. In particular, Ex4-C16 in DA-GCS hydrogels was found to be present around the injection site up to 10 days after subcutaneous administration, whereas Ex4 in DA-GCS hydrogels was cleared from injection sites in ∼2 days in Institute of Cancer Research (ICR) mice. Finally, the hypoglycemia induced by Ex4-C16 DA-GCS hydrogels was maintained for >7 days. It demonstrates that Ex4-C16 DA-GCS hydrogels offer a potential delivery system for the long-term treatment of type 2 diabetes.

Cheng et al. (2014) developed an injectable thermosensitive chitosan/gelatin/glycerol phosphate (CS/G/GP) hydrogel as a sustained-release system of latanoprost for glaucoma treatment. The latanoprost-loaded CS/G/GP hydrogel can gel within 1 min at 37 °C, and it showed a good *in vitro* and *in vivo* biocompatibility. The results prove a sustained release of latanoprost from CS/G/GP hydrogel. A rabbit model of glaucoma was established by intravitreal injection of triamcinolone acetonide. After a single subconjunctival injection of latanoprost-loaded CS/G/GP hydrogel, intraocular pressure (IOP) was significantly decreased within 8 days and then remained at a normal level. It suggests that latanoprost-loaded CS/G/GP hydrogel might have a potential application in glaucoma therapy.

Yadollahi et al. (2015) synthesized antibacterial chitosan/silver bio-nanocomposite hydrogel beads for drug delivery. Kono & Teshirogi (2015) used the cyclodextrin-grafted chitosan hydrogels for controlled drug delivery. Yang et al. (2013) developed the pH-sensitive interpenetrating network hydrogels based on chitosan derivatives and alginate for oral drug delivery.

## 3. Chitosan and its derivatives as vehicles to deliver gene or protein

Chitosan is a good choice for gene delivery systems because it has the cation that can be effectively bound to an anion-containing DNA and protect DNA from nuclease degradation. Moreover, chitosan particles loaded with DNA are more stable at the time of storage, so it is of great significance to use chitosan as a vector for gene drug (Duceppe & Tabrizian, 2010). Chitosan as a protein vehicle can protect protein drugs from enzyme degradation and can control the release of drugs to achieve the purpose of sustained or controlled release, which can help extend the biological activity of protein drugs *in vivo* (Lü et al., 2010).

The low solubility of chitosan has limited its gene transfection efficiency. The water solubility of chitosan can be improved by appropriate derivatization (Siafaka et al., 2015). Toh et al. (2011) reported the synthesis of different substitution degrees of succinated chitosans (CS-succ) to increase water solubility. It indicated that the plasmid DNA was readily entrapped at a CS-succ/DNA weight ratio of 20; the sizes and zeta potentials were between 110–140 nm and ±1–5 mV, and the polyplexes exhibited low cytotoxicity against HEK 293 T cells. CS-succ with 5 and 10% degrees of substitution showed improved transfection efficiency as compared with nascent chitosan.

Rajesh et al. (2012) synthesized and evaluated the LCS-*g*-PEI (lauryl CS graft polyethyleneimine) polymer as a potential vehicle of therapeutic molecules, such as the p53 gene and DOX. This polymer had lower interactions with blood components than the unmodified PEI. LCS-g-PEI buffered protons, which were protected DNA from nuclease attack, induced effective gene transfection efficiency in the C6 cell line. LCS-g-PEI, which had incorporated DOX, exhibited an enhanced release of this compound at pH 5. It demonstrated that LCS-g-PEI had efficacy in the enhancement of drug uptake and the promotion of gene expression in the C6 cell line. So, LCS-g-PEI shows great promise as a drug/gene vehicle with potential applications in cancer therapy.

Chitosan-disulfide-conjugated low molecular weight polyethylenimine (LMW-PEI) (CS-ss-PEI) was prepared by conjugating LMW-PEI to chitosan through oxidization of thiols introduced for the formation of disulfide linkage (Zhao et al., 2013). With the increment in the LMW-PEI component, the copolymer showed increased DNA binding ability and formed denser nanocomplexes. CS-ss-PEI exhibited low cytotoxicity in COS-1, HepG2 and 293 T cells over the different weight ratios. The transfection efficiency of CS-ss-PEI4 was significantly higher than that of PEI25k and comparable with Lipofectamine in mediating luciferase expression. Its application for bone morphogenetic protein (BMP2) gene delivery was confirmed in C2C12 cells by BMP2 expression. For inducing *in vitro* osteogenic differentiation, CS-ss-PEI4 mediated BMP2 gene delivery showed a stronger effect in MG-63 osteoblast cells and stem cells in terms of alkaline phosphatase activity and mineralization compared with PEI25k and Lipofectamine. Overall, it provided a potential gene delivery system for orthopedic-related disease.

The successful clinical translation of small interference RNA (siRNA)-based therapeutics requires efficient vehicle systems that can specifically deliver siRNA within the cytosol of the target cells. Although numerous polymeric nanocarriers forming ionic complexes with siRNA have been investigated for cancer therapy, their poor stability and lack of tumor targetability have hindered their *in vivo* applications (Forbes & Peppas, 2014). For chitosan, derivatization can improve its stability and targeting ability, for example, Hu et al. (2016) used the chitosan-based glycolipid-like nanocarrier for selective redox-responsive siRNA delivery.

Wassmer et al. (2013) investigated the release kinetics of chitosan MPs (CSMPs) *in vitro*, and assesses their biocompatibility and cytotoxicity on retinal cells *in vitro* and *in vivo*. The *in vitro* release kinetics were dependent on the protein encapsulated, with bovine serum albumin (BSA) showing higher release than tat-EGFP (enhanced green fluorescent protein fused to the transactivator of transcription peptide). CSMPs, which contained the encapsulated tat-EGFP, were tested for cellular toxicity in photoreceptor-derived 661 W cells. They showed no signs of *in vitro* cell toxicity at a low concentration (up to 1 mg/mL), but they were associated with cytotoxic effects at a higher concentration (10 mg/mL). *In vivo*, CSMPs, which were injected into the subretinal space, were found beneath the photoreceptor layer of the retina, and persisted for at least 8 weeks. Similar to the *in vitro* studies, the lower concentration of CSMPs was generally well tolerated, but the higher concentration resulted in cytotoxic effects and in reduced retinal function. In a word, it was suggested that CSMPs were effective long-term delivery agents to the retina, but the concentration of chitosan might affect cytotoxicity.

Liu et al. (2010) used the calcium-carboxymethyl chitosan hydrogel beads to deliver protein. Katas et al. (2013) developed the chitosan nanoparticles for protein/siRNA delivery. Ayensu et al. (2012) prepared the lyophilized chitosan wafers for protein drug delivery via the buccal mucosa.

## 4. Conclusion

Chitosan, as a biodegradable polymer with excellent properties, is a promising new excipient for pharmaceutical preparations. With the deepening of research on chitosan and its derivatives, the organic combination of chitosan bioactivities and drugs will promote the development of drug preparations, and be widely used in the new dosage forms of drugs. The development of chitosan vehicles that can control the release of drug will become one of the future research directions.

## References

[CIT0001] Alishahi A, Mirvaghefi A, Tehrani MR, et al. (2011). Chitosan nanoparticle to carry vitamin C through the gastrointestinal tract and induce the non-specific immunity system of rainbow trout (*Oncorhynchus mykiss*). Carbohydrate Polym 86:142–6.

[CIT0002] Anitha A, Maya S, Deepa N, et al. (2012). Curcumin-loaded *N*,*O*-carboxymethyl chitosan nanoparticles for cancer drug delivery. J Biomater Sci Polym Ed 23:1381–400. 21722423 10.1163/092050611X581534

[CIT0003] Ayensu I, Mitchell JC, Boateng JS. (2012). Development and physico-mechanical characterisation of lyophilised chitosan wafers as potential protein drug delivery systems via the buccal mucosa. Colloids Surf B: Biointerf 91:258–65. 10.1016/j.colsurfb.2011.11.004 22130527

[CIT0004] Bhargava M, Bhargava S, Jain S, Jain R. (2015). P032: development and characterization of surface modified chitosan nanoparticles for selective targeting of lamivudine to hepatocyte. J Viral Hepatitis 22:35.

[CIT0005] Bhattarai N, Gunn J, Zhang M. (2010). Chitosan-based hydrogels for controlled, localized drug delivery ⋆. Adv Drug Deliv Rev 62:83–99. 19799949 10.1016/j.addr.2009.07.019

[CIT0006] Calderón L, Harris R, Cordoba-Diaz M, et al. (2013). Nano and microparticulate chitosan-based systems for antiviral topical delivery. Eur J Pharmaceut Sci 48:216–22. 10.1016/j.ejps.2012.11.002 23159663

[CIT0007] Cheng YH, Hung KH, Tsai TH, et al. (2014). Sustained delivery of latanoprost by thermosensitive chitosan-gelatin-based hydrogel for controlling ocular hypertension. Acta Biomater 10:4360–6. 24914827 10.1016/j.actbio.2014.05.031

[CIT0008] Duceppe N, Tabrizian M. (2010). Advances in using chitosan-based nanoparticles for in vitro and in vivo drug and gene delivery. Expert Opin Drug Deliv 7:1191–207. 20836623 10.1517/17425247.2010.514604

[CIT0009] Feng C, Wang Z, Jiang C, et al. (2013). Chitosan/*O*-carboxymethyl chitosan nanoparticles for efficient and safe oral anticancer drug delivery: *In vitro* and *in vivo* evaluation. Int J Pharmaceut 457:158–67. 10.1016/j.ijpharm.2013.07.079 24029170

[CIT0010] Forbes DC, Peppas NA. (2014). Polymeric nanocarriers for sirna delivery to murine macrophages. Macromol Biosci 14:1096–105. 24753265 10.1002/mabi.201400027

[CIT0011] Gratieri T, Gelfuso GM, Freitas OD, et al. (2011). Enhancing and sustaining the topical ocular delivery of fluconazole using chitosan solution and poloxamer/chitosan *in situ* forming gel. Eur J Pharm Biopharm 79:320–7. 21641994 10.1016/j.ejpb.2011.05.006

[CIT0012] Grenha A, Gomes ME, Rodrigues M, et al. (2010). Development of new chitosan/carrageenan nanoparticles for drug delivery applications. J Biomed Mater Res A 92:1265–72. 19322874 10.1002/jbm.a.32466

[CIT0013] Hu Y, Liu N, Tan Y, Hu F. (2016). Selective redox-responsive siRNA delivery mediated by chitosan based glycolipid-like nanocarrier. Nanomedicine 12:549–50.

[CIT0014] Katas H, Raja MAG, Kai LL. (2013). Development of chitosan nanoparticles as a stable drug delivery system for protein/siRNA. Int J Biomater 2013:146320. 24194759 10.1155/2013/146320 PMC3806166

[CIT0015] Kawadkar J, Jain R, Kishore R, et al. (2013). Formulation and evaluation of flurbiprofen-loaded genipin cross-linked gelatin microspheres for intra-articular delivery. J Drug Target 21:200–10. 23249324 10.3109/1061186X.2012.745549

[CIT0016] Khodadust R, Unsoy G, Gunduz U. (2014). Development of poly (I:C) modified doxorubicin loaded magnetic dendrimer nanoparticles for targeted combination therapy. Biomed Pharmacother 68:979–87. 25458787 10.1016/j.biopha.2014.10.009

[CIT0017] Kono H, Teshirogi T. (2015). Cyclodextrin-grafted chitosan hydrogels for controlled drug delivery. International Int J Biol Macromol 72:299–308. 10.1016/j.ijbiomac.2014.08.030 25192852

[CIT0018] Lam PL, Lee KK, Wong RS, et al. (2012). Development of hydrocortisone succinic acid/and 5-fluorouracil/chitosan microcapsules for oral and topical drug deliveries. Bioorg Med Chem Lett 22:3213–18. 22460032 10.1016/j.bmcl.2012.03.031

[CIT0019] Lee BR, Oh KT, Baik HJ, et al. (2010). A charge-switched nano-sized polymeric carrier for protein delivery. Int J Pharmaceut 392:78–82. 10.1016/j.ijpharm.2010.03.028 20298771

[CIT0020] Lee JH, Lee CK, Bae SH, et al. (2011). Preparation and characterization of self-assembled glycol chitosan hydrogels containing palmityl-acylated exendin-4 for extended hypoglycemic action. J Pharmaceut Investig 41:173–8.

[CIT0021] Liu Z, Jiao Y, Zhang Z. (2010). Calcium-carboxymethyl chitosan hydrogel beads for protein drug delivery system. J Appl Polym Sci 103:3164–8.

[CIT0022] Lü S, Liu M, Ni B. (2010). An injectable oxidized carboxymethylcellulose/n-succinyl-chitosan hydrogel system for protein delivery. Chem Eng J 160:779–87.

[CIT0023] Mei LT, Choong PFM, Dass CR. (2009). Review: doxorubicin delivery systems based on chitosan for cancer therapy. J Pharm Pharmacol 61:131–42. 19178759 10.1211/jpp/61.02.0001

[CIT0024] Mengatto LN, Helbling IM, Luna JA. (2012). Recent advances in chitosan films for controlled release of drugs. Recent Pat Drug Deliv Formul 6:156–70. 22436027 10.2174/187221112800672967

[CIT0025] Millotti G, Perera G, Vigl C, et al. (2011). The use of chitosan-6-mercaptonicotinic acid nanoparticles for oral peptide drug delivery. Drug Deliv 18:190–7. 21039318 10.3109/10717544.2010.522611

[CIT0026] Mouez MA, Zaki NM, Mansour S, Geneidi AS. (2014). Bioavailability enhancement of verapamil HCl *via* intranasal chitosan microspheres. Eur J Pharmaceut Sci 51:59–66. 10.1016/j.ejps.2013.08.029 23999035

[CIT0027] Patil SB, Sawant KK. (2011). Chitosan microspheres as a delivery system for nasal insufflation. Colloids Surf B Biointerfaces 84:384–9. 21320767 10.1016/j.colsurfb.2011.01.030

[CIT0028] Pengpong T, Sangvanich P, Sirilertmukul K, Muangsin N. (2014). Design, synthesis and *in vitro* evaluation of mucoadhesive *p*-coumarate-thiolated-chitosan as a hydrophobic drug carriers. Eur J Pharm Biopharm 86:487–97. 24333665 10.1016/j.ejpb.2013.11.009

[CIT0029] Rajesh R, Rekha MR, Sharma CP. (2012). Evaluation of lauryl chitosan graft polyethyleneimine as a potential carrier of genes and anticancer drugs. Process Biochem 47:1079–88.

[CIT0030] Rani M, Agarwal A, Negi YS. (2010). Chitosan based hydrogel polymeric beads - as drug delivery system. Bioresources 5:2765–807.

[CIT0031] Riva R, Ragelle H, Rieux AD, et al. (2011). Chitosan and chitosan derivatives in drug delivery and tissue engineering. Adv Polym Sci 244:19–44.

[CIT0032] Saranya N, Moorthi A, Saravanan S, et al. (2011). Chitosan and its derivatives for gene delivery. Int J Biol Macromol 48:234–8. 21134396 10.1016/j.ijbiomac.2010.11.013

[CIT0033] Saravana KA, Ramaswamy NM. (2011). Chitosan microspheres as potential vaccine delivery systems. Int J Drug Del 3:43–50.

[CIT0034] Sezer AD, Cevher E. (2012). Topical drug delivery using chitosan nano- and microparticles. Expert Opin Drug Deliv 9:1129–46. 22769587 10.1517/17425247.2012.702752

[CIT0035] Shen JM, Tang WJ, Zhang XL, et al. (2012). Novel carboxymethyl chitosan-based folate/Fe_3_O_4_/CdTe nanoparticle for targeted drug delivery and cell imaging. Carbohydrate Polym 88:239–49.

[CIT0036] Siafaka PI, Titopoulou A, Koukaras EN, et al. (2015). Chitosan derivatives as effective nanocarriers for ocular release of timolol drug. Int J Pharmaceut 495:249–64. 10.1016/j.ijpharm.2015.08.100 26341322

[CIT0037] Sonia TA, Sharma CP. (2011). Chitosan and its derivatives for drug delivery perspective. Adv Polym Sci 243:23–53.

[CIT0038] Supper S, Anton N, Boisclair J, et al. (2014). Chitosan/glucose 1-phosphate as new stable *in situ* forming depot system for controlled drug delivery. Eur J Pharm Biopharm 88:361–73. 24859306 10.1016/j.ejpb.2014.05.015

[CIT0039] Termsarasab U, Yoon IS, Park JH, et al. (2014). Polyethylene glycol-modified arachidyl chitosan-based nanoparticles for prolonged blood circulation of doxorubicin. Int J Pharmaceut 464:127–34. 10.1016/j.ijpharm.2014.01.015 24451239

[CIT0040] Toh EK, Chen HY, Lo YL, et al. (2011). Succinated chitosan as a gene carrier for improved chitosan solubility and gene transfection. Nanomedicine 7:174–83. 20732455 10.1016/j.nano.2010.07.007

[CIT0041] Umadevi SK, Thiruganesh R, Suresh S, Reddy KB. (2010). Formulation and evaluation of chitosan microspheres of aceclofenac for colon-targeted drug delivery. Biopharm Drug Dispos 31:407–27. 20848388 10.1002/bdd.722

[CIT0042] Varshosaz J. (2007). The promise of chitosan microspheres in drug delivery systems. Expert Opin Drug Deliv 4:263–73. 17489653 10.1517/17425247.4.3.263

[CIT0043] Vivek R, Babu VN, Thangam R, et al. (2013). Ph-responsive drug delivery of chitosan nanoparticles as tamoxifen carriers for effective anti-tumor activity in breast cancer cells. Colloids Surf B Biointerf 111:117–23. 10.1016/j.colsurfb.2013.05.018 23787278

[CIT0044] Wang C, Mallela J, Garapati US, et al. (2013). A chitosan-modified graphene nanogel for noninvasive controlled drug release. Nanomedicine 9:903–11. 23352802 10.1016/j.nano.2013.01.003 PMC3783966

[CIT0045] Wang J, Chen JS, Zong JY, et al. (2010). Calcium carbonate/carboxymethyl chitosan hybrid microspheres and nanospheres for drug delivery. J Phys Chem C 114:18940–5.

[CIT0046] Wassmer S, Rafat M, Fong WG, et al. (2013). Chitosan microparticles for delivery of proteins to the retina. Acta Biomater 9:7855–64. 23623991 10.1016/j.actbio.2013.04.025

[CIT0047] Xing ZH. (2013). Preparation and characterization of folate modified-chitosan nanoparticles as new tumor-targeting drug carrier. Asian J Chem 25:9532–6.

[CIT0048] Xing ZH. (2013). Preparation and characterization of folate-decorated chitosan microspheres as a sustained-release drugs carrier. Amr 684:57–62.

[CIT0049] Yadollahi M, Farhoudian S, Namazi H. (2015). One-pot synthesis of antibacterial chitosan/silver bio-nanocomposite hydrogel beads as drug delivery systems. Int J Biol Macromol 79:37–43. 25931399 10.1016/j.ijbiomac.2015.04.032

[CIT0050] Yang J, Chen J, Pan D, et al. (2013). pH-sensitive interpenetrating network hydrogels based on chitosan derivatives and alginate for oral drug delivery. Carbohydrate Polym 92:719–25. 10.1016/j.carbpol.2012.09.036 23218359

[CIT0051] Zhang J, Tang Q, Xu X, Li N. (2013). Development and evaluation of a novel phytosome-loaded chitosan microsphere system for curcumin delivery. Int J Pharm 448:168–74. 23524117 10.1016/j.ijpharm.2013.03.021

[CIT0052] Zhao X, Li Z, Pan H, et al. (2013). Enhanced gene delivery by chitosan-disulfide-conjugated LMW-PEI for facilitating osteogenic differentiation. Acta Biomater 9:6694–703. 23395816 10.1016/j.actbio.2013.01.039

[CIT0053] Zhou N, Zan X, Wang Z, et al. (2013). Galactosylated chitosan-polycaprolactone nanoparticles for hepatocyte-targeted delivery of curcumin. Carbohydrate Polym 94:420–9. 10.1016/j.carbpol.2013.01.014 23544558

